# Minimization of Male Suffering: Social Perception of Victims and Perpetrators of Opposite-Sex Sexual Coercion

**DOI:** 10.1007/s13178-016-0226-0

**Published:** 2016-03-19

**Authors:** Anna Magda Studzinska, Denis Hilton

**Affiliations:** 1 Université Toulouse Jean Jaurès, 5, Allées Antonio Machado, F-31058 Toulouse Cedex 9, France; 2 SWPS University of Social Sciences and Humanities, Campus in Sopot, Polna 16/20, 81-745 Sopot, Poland

**Keywords:** Social perception of suffering, Victim perception, Perpetrator perception, Sexual harassment, Sexual coercion

## Abstract

Studies show equal impact of sexual harassment (SH) on men and women, whereas lay perceptions are that women suffer more. We identify the phenomenon of minimization of male suffering (MMS), which occurs when people assume that SH has less effect on men’s well-being and which results in the perpetrators of SH on men being evaluated less harshly. To verify whether these effects occur, we conducted two studies in which we presented stories describing acts of sexual coercion (SC, study 1) and SC or financial coercion (FC, study 2) and measured the perceived suffering of victims and the perception of the perpetrators. Both studies showed that female victims were perceived to suffer more from SC and FC and that perpetrators of both acts on women were evaluated more negatively. The results support our hypothesis that the suffering of male victims is minimized as they are perceived to suffer less than women.

## Introduction

Sexual coercion (SC) is considered to constitute the most common form of sexual harassment (SH), independently of the sex of the evaluator (Rotundo et al. 2001), and of the sex of the perpetrator or the victim (Runtz and O’Donell 2003). In the studies presented in this paper, we focus on the perceived consequences of this kind of SH. Despite research suggesting that male and female victims of SH suffer equal distress (Street et al. 2007; Vogt et al. 2005; Magley et al. 1999), we propose that people see women as suffering more from SH than men, leading to a minimization of male suffering (MMS). This occurs when men who became victims of SH are thought to suffer less from its consequences and when their perpetrators are evaluated less negatively than those who sexually harass women.

## The Prevalence and Consequences of Sexual Harassment

SH is unwelcomed behavior related to one’s gender and one’s sexuality that is perceived by the recipient as unpleasant and that causes the recipient to feel psychological distress (Gelfand et al. 1995). In their model of SH, based on empirical data, Fitzgerald et al. (1995) identify three types of SH. The most frequent type which is also assumed to be the least hurtful is gender harassment; this entails verbal and nonverbal behaviors that insult or degrade a person because of his or her sex. The second type of SH, i.e., unwanted sexual attention, involves unwanted sexual advances, including unwelcomed touching or constant attempts at establishing an intimate or sexual relationship. Finally, sexual coercion involves threats and/ or promises that the target will be granted extra opportunities (e.g., promotion) or s/he will be spared from negative events (e.g., will not be fired) if s/he engages in a sexual relationship with the perpetrator. Research on effects of all types of SH shows that its victims suffer from numerous psychological and somatic problems, which include, but are not limited to, depression, anxiety, post-traumatic stress disorder (PTSD) symptoms, headaches, and decrease in sleep or weight loss (Pina and Gannon 2012; Willness et al. 2007; Charney and Russell 1994).

In addition, these effects are not limited to women. It should be noted that even though SH is more frequently experienced by women, men are not only victims of SH but the number of claims of SH of men is also increasing (Foote and Goodman-Delahunty 2005). Several studies show men of different ages and backgrounds to be victims of different types of SH. For example, in a study by Kearney and Rochlen (2011) on male college students, 73.7 % of Mexican-American students and 84.4 % of Caucasian students were found to have experienced SH. Settles et al. (2011) found that among the US armed service personnel, 19.4 % of males (*n* = 1764) and 51.7 % of females (*n* = 4540) have experienced some forms of SH. In a Norwegian employee sample, it was found that 18.4 % experienced some forms of SH, while 0.9 % of men and 0.8 % of women stated that they had experienced SC (i.e., had been asked to have sex in order to avoid something or to gain something; Birkeland et al. 2010). Overall, SH is a widespread problem that, depending on the estimations, affected or will affect from 30 to 50 % of women and from 10 to 15 % of men (Charney and Russell 1994; Directorate-General for Employment, Industrial Relations and Social Affairs Unit V/D.5 1998). Evidently, the prevalence of different types of SH is not the same. For example, a study on 208 employed female students (Hitlan et al. 2006) showed that 70 % of them experienced gender harassment and 53 % experienced unwanted sexual attention. Leskinen et al. (2011) showed that women in the military and female lawyers experience gender harassment in the absence of other types of SH more often than gender harassment with unwanted sexual attention or just unwanted sexual attention or sexual coercion. However, they showed that even the instances of “just” gender harassment are related in a negative way to women’s psychological well-being and health and to increased stress levels. A study on 2319 female and 1627 male former reservists (Street et al. 2007) showed that 72.8 % of women and 42.0 % of men experienced one of the forms of SH, with lewd comments being the most common, followed by negative gender related remarks, unwanted sexual attention, and sexual coercion as the least common form of SH. American Department of Defense’s SH study (Department of Defense 2013) on 63,177 male and 45,301 female active duty members showed that overall, 23 % of women and 4 % of men experienced SH in the past 12 months. Forty-one percent of women and 20 % of men experienced gender harassment, 23 % of women and 5 % of men experienced unwanted sexual attention, and 8 % of women and 2 % of men experienced sexual coercion.

SH appears to be also prevalent at the workplace in Poland, where the presented studies were carried out. According to a study conducted by Public Opinion Research Center (Centrum Badania Opinii Społecznej 2007) on 424 employees, 22 % of the participants witnessed lewd comments, 7 % experienced or witnessed unwanted sexual attention from their colleagues and 4 % from their supervisors, and 2 % declared knowing of a situation when a person benefited from having a sexual relationship with their supervisor. Moreover, 5 % of men and 13 % of women claimed to have been victims of lewd comments and that every 20th woman experienced unwanted sexual attention, while “men [constitute] just a few cases” (p. 8). Furthermore, 36 % of Polish high school age students experienced some forms of sexual violence and it seems that adolescents are more likely to be victims of SH than adults (Izdebski 2012). A study on SH in public places showed that 85 % of women and 44 % of men (*n* = 818; 72 men) experienced it at least once and that both Polish men and women agree on what constitutes SH (Gober and Roszak 2012). A newer study on the prevalence of SH in Poland (Sulej and Jablonska 2015; Roszak 2015) showed that when asked if they ever experienced “unwanted courtship, erotic provocations, or sexual proposals,” 11.3 % of women and 6.8 % of men answered “often” or “a few times” and 12.7 % of women and 14.7 % of men answered rarely.

Waldo et al. (1998) studied the SH of men and found that they evaluated lewd comments as less upsetting than unwanted sexual attention (no men in their samples experiences sexual coercion). Similarly, Gerrity (2000) compared the outcomes of gender harassment and unwanted sexual attention among male university employees and showed that the latter had a stronger negative impact on the emotional health, depression, anxiety levels, or self-esteem. However, a study by Langhout et al. (2005) showed how the frequency with which different types of SH occur can influence the level of stress that it causes. For example, unwanted sexual attention seems to be equally stressful whether it occurred once or multiple times, while the level of stress increases with the number of gender harassment (lewd comments and negative gender-related remarks) experiences.

Research results suggest that experience of SH has equal impact on men’s and women’s psychological health and in some cases has a greater impact on men’s well-being. A meta-analysis carried out by Willness et al. (2007) showed that SH is linked to numerous mental health problems (such as anxiety, depression, and negative mood), decreased well-being, and increased PTSD levels; it is also related to such physical health symptoms such as nausea, headaches, shortness of breath, or exhaustion. As shown by Birkeland et al. (2010), it is not one’s sex that influences the impact SH has on a person but the very fact of being a SH victim. Both men and women who experienced frequent and explicit SH were found to have a higher number of mental health problems, compared to those who did not experience it at all. In addition, male victims of SH experienced lower job satisfaction than female victims. Among former reservists, in a model with participants’ sex, SH, and interaction of the two as predictors, SH was found to be the strongest predictor of depression, and at higher levels of SH, it was the men that reported more depression symptoms and worse overall mental health. Additionally, when cases of sexual assault were removed, male SH victims were found to experience higher levels of PTSD than women. Moreover, men who experienced SH were the most likely to suffer from PTSD, depression, and mental health problems (Street et al. 2007a, b). Similarly, in a sample of Gulf War I veterans, the intensity of both depression and anxiety rose more sharply for men than for women with the increase of SH levels (Vogt et al. 2005). Another study on military personnel showed a positive linear relationship between the frequency of SH and psychological and physiological problems among both men and women (Magley et al. 1999). Men and women who found different types of SH (gender harassment, unwanted sexual attention, and sexual coercion) to be frightening experienced similar levels of distress (Settles et al. 2011), additionally, for men the perception of SH as bothersome that lead to their heightened intensity of distress. In a different study by the same team (Settles et al. 2014), the relationship between frightening appraisals of SH acts and psychological distress was stronger for men than women.

Street (2009) claims that SH can have different negative impact on men and women. Men who experienced sexual trauma are more at risk for mental health problems, they experience strong feelings of shame and self-blame, and can suffer from substance abuse. Analyzing the medical records of American Army veterans, Kimerling et al. (2007) showed that experiences of military sexual trauma (which includes sexual harassment and sexual assault) are strongly linked to mental and psychical health problems among men and women. Both men and women are at a high risk for dissociative, eating, and depression disorders. A PTSD diagnosis, alcohol problems, and anxiety problems can also happen to both men and women but seem to be more likely among women. Adjustment disorders, bipolar disorders, schizophrenia, and psychosis are more common among men. It should be noted that the majority of samples used in SH research comes from military populations, among which, according to popular stereotypes, men might be expected not to fold under the pressure of SH. However, Willness et al. (2007) used the military vs civilian status as a potential moderator in a meta-analysis of the consequences of SH, and the results suggest it to be a significant only in case of work satisfaction. Other SH outcome variables, such as coworker satisfaction, or organizational commitment, mental health, and physical health were not influenced by the military vs civilian status.

Overall, male victims of SH are often found to experience negative outcomes of SH equally or more severe than their female colleagues. All in all, it seems that while women suffer more frequently from SH in their work and private life, when SH occurs and is of equivalent intensity, it affects men and women at least to the same extent, causing mental health and physical problems of similar severity and in some cases causing the men to suffer more than women (e.g., Street et al. 2007a, b; Magley et al. 1999).

### The Perception of the Consequences of Sexual Harassment

In contrast to the evidence presented above, the literature on the ordinary person’s perception of SH suggests that women’s experience of SH is seen as more traumatic than men’s. There are studies that assess to what degree the given behavior can be perceived as harassing (LaRocca and Kromrey 1999) or how upset the victim of gender harassment and unwanted sexual attention might be (McKinney 1992). These studies show that male victims are perceived to be less harassed, less upset, and less injured. In other studies, the participants are asked to imagine how they would feel if they should become victims of SH. Compared to men, women assumed they might be more anxious and especially so when the type of SH in question is sexual coercion (Berdahl et al. 1996). When asked to imagine that they had been asked directly by a colleague of the opposite sex if they want to have sexual relationship with them, the majority of men thought they would be flattered, while the majority of women thought they would be offended (Konrad and Gutek 1986). In comparison to men, women also thought that they would be affected more should they be touched on their genitalia by an acquaintance (Struckman-Johnson and Struckman-Johnson 1993). This shows a clear pattern, indicating that the perceived effect SH has on its male victims is minimized whether the evaluation is aimed at a hypothetical victim or at oneself in a hypothetical victimizing situation.

### The Present Study

Given the mismatch between the reality and the perception of the effects of SH on men, we seek to better understand how male and female victims and perpetrators of SH are perceived. The presented research adds to the literature, as the previously mentioned studies do not pay detailed attention to perception of SH victims’ distress, nor to how these victims are perceived to experience the event, nor to evaluation of the characteristics of male and female victims of SH. Overall, we focused on analyzing what is the social perception of SH victims’ suffering, which was not done thus far in such detail, and what is the evaluation of the SH perpetrators, which, to the best of our knowledge, has not been analyzed. Moreover, we did not find any studies concerning perceptions of SH or its victims and perpetrators conducted in Poland.

To verify the perceived level of psychological suffering (perception of emotions) and of victims’ reactions to the event, we use variables commonly used in research on SH victims, such as depression, anxiety, somatic symptoms, or emotions connected to the event with the use of measures often used in studies concerning actual experiences of SH victims, thus eliminating a potential source of a discrepancy between lay and scientific perceptions of victim’s suffering. We also study the evaluation of the perpetrator’s personal characteristics which was not done so far, to the best of our knowledge. For the evaluation of perpetrators (person evaluation), we use variables typically used in social perception research, such as communion and agency characteristics (also labeled as morality and competence or socially and intellectually good–bad traits), which have been established by numerous studies (e.g., Wojciszke 1997) to constitute the two main dimensions of social judgment. Communion is an especially important dimension when evaluating others and agency when judging oneself. Additionally, the fact that somebody is liked depends mostly on how communal s/he is perceived to be, while evaluation of a person’s agency influences the level of respect s/he deserves (Wojciszke et al. 2009). As such, low evaluation on any of the dimensions (communion, agency, liking, and respect) constitutes negative evaluation of a person.

We therefore decided to explore the perceived consequences of SH for male and female victims, as well as the evaluation of the perpetrator. Taking into consideration that men and women experience SH and are influenced by it to a similar extent, yet the social perception of male and female victims of SH seems to differ, we hypothesize that there exists a minimization of male suffering effect. As such, we put forward the following hypotheses:

1. Male victims of SH are perceived to suffer less psychological damage from SH (studies 1 and 2).
2. Perpetrators of SH on female victims are evaluated more negatively than perpetrators of SH on male victims (studies 1 and 2).
3. Male victims are perceived to see SH in less negative way than female victims (study 2).

Moreover, the previously described studies do not compare SH to other types of assault, thus providing information on SH but not showing a wider perspective from which we could learn whether female victims are perceived to suffer less than male victims only in case of SH or if it is a more general phenomenon. For this reason, in study 2, we compare the evaluation of victim’s suffering and the evaluation of the perpetrator in case of SC and in case of a different type of coercion, namely, a financial one, to answer the question if the MMS effect is specific to SH or does it occur in other types of assaults.

## Method

**Study 1. The effects of victim’s sex on perceptions of his/her suffering and on the characteristics of the perpetrator in opposite-sex sexual coercion**

### Materials and Procedure

The sample included 154 participants (37 men and 117 women) recruited through the internet. We posted an invitation to an online study on multiple Polish public internet forums (for example, different forums of major Polish cities), started an open event on social media, and asked the potential participants to also share the invitation with their friends and family. Due to a large difference in the number of men and women in the sample, we decided to analyze the two groups separately. Mean age for the whole group was 36.77 (SD = 14.00). The majority (76.6 %) had higher education, 20 % had high school education, and the rest of the participants had either primary or vocational education. Mean age among women was 37.43 years (SD = 12.83) and among men 35.72 (SD = 16.33); the distribution of education for men and women separately was the same as for the whole group.

The study was carried out in Polish. We obtained Ethics Committee approval to conduct the described research. In the first part of the study, the participants were asked to state their sex, age, and education. Next, they were randomly assigned to read one of two vignettes which described a young person (a man or a woman) during an internship. In one version, the supervisor was male and the intern was female (M on F), and in the second one, the supervisor was female and the intern was male (F on M). The intern knew s/he might be hired after the internship and when the decision day came, as his/her supervisor informed him/her that s/he will be offered a permanent job if s/he agrees to have sex with the supervisor. Subsequently, the participants were asked to fill out one of the measures indicating how the event influenced the victim’s well-being. Because the measures we used are long, each participant was assigned randomly to one of four subgroups to fill out one measure used to evaluate the perceived distress of the victim. As this was a first study from a planned larger project, we were looking to learn which symptoms of human suffering differentiate the best between male and female victims; thus, we decided to use and test different outcome variables that measure depression, anxiety, or somatic symptoms. Dividing the sample into four subgroups lowered the statistical power of the results and created very small subsamples (especially of male participants), but this way, we were hoping to gain a more detailed insight into how victim’s suffering is perceived.

In order to test perceived negative impact of the event on the victim, we measured how the participants perceive the victim’s depression, anxiety, somatic symptoms, and overall well-being levels. In order to do so, we used changed versions of clinical tools usually used to measure depression, anxiety, somatic symptoms, and well-being. Namely, instead of asking to evaluate how they feel, we asked them to evaluate how the described intern feels.

To measure the perceived depression of the victim, the first group received an inventory based on a modified version of the *Beck Depression Inventory* (BDI). The scale consisted of 21 items, each describing a depressive symptom with four levels of intensity. The answers in the inventory range from 0 (*does not have this symptom at all*) to 3 (*high severity*); i.e., *She does not feel sad* (*0*) and *He is so sad or unhappy that he cannot stand it* (3). The items were changed so that the participants were not responding to how they themselves feel but rather how the described person feels. The participants were asked to estimate to what degree did they think the intern described in the vignette experienced each of the symptoms. The final score was obtained by adding points of all the items. Thirty-eight women and seven men filled out this scale; Cronbach’s alpha for female participants was 0.89 and for male participants 0.67.

To measure the perceived anxiety, depression, and somatic symptoms, the second group received a modified version of the *Hopkins Symptom Checklist-25* (HSCL). The original scale was adjusted so that the participants responded to the perceived influence of the event on the intern described in the vignette. Examples of items used in the study include *She experiences spells of terror or panic* (anxiety; 10 items); *He feels low in energy, slowed down* (depression; 13 items); and *She has poor appetite* (somatic; 2 items). Each item was scored on a 1 (*not at all*) to 5 (*extremely*) scale, and for each of the subscales, the average was computed. Twenty-seven women and nine men filled out this scale; Cronbach’s alpha among women for anxiety was 0.96, for depression 0.99, and for somatic symptoms 0.60 and for men (respectively) were 0.97, 0.99, and 0.56.

In order to measure the perceived well-being of the victim, the third group received a modified version of the *WHO-5 Well-Being Index* (WHO)—a short version of WHO Well-Being Questionnaire. It consisted of five items concerning positive well-being (two items), energy (two items), and anxiety (one item; reversed scoring); once again, the items were rephrased, so that the participants’ responses concerned the described person’s well-being. Examples are *He is happy, satisfied, or pleased with personal life* and *She is energetic, active, and vigorous*. Each statement was scored on a six-point scale, ranging from *all the time* (5) to *never* (0). The result was obtained by adding up the scores for all items, giving possible range of 0 to 25 and with a high score being indicative of high well-being. Thirty-two women and eight men filled out this scale; Cronbach’s alpha was 0.96 for the female and 0.92 for the male sample. We also used the *Mississippi PTSD Scale-Civilian* (PTSD), but due to an error of the website used to distribute the study, the scores were not recorded, and as such, we do not have the results for this scale.

As studies show, morality and competence constitute two basic dimensions of social judgment (cf. Wojciszke 2005). Those dimensions are important in the process of evaluation, as when we meet new people, we must instantly establish if their intentions toward us are good or bad (morality) and if they are able to carry them out (competence). Moreover, we like those who we find moral and respect and those who we find competent (Wojciszke et al. 2009).

We used a scale to measure the perceived morality and competence, as well as liking and respect of others that was previously used in research on differences in gender perception in Poland (e.g., Kosakowska 2006). All of the participants responded on a scale from 1 to 7 to a list of eight adjectives to evaluate the perpetrator’s perceived communion, agency, liking, and respect. Cronbach’s alphas for male and female participants for this measure were relatively low (the majority around 0.51); consequently, we decided to analyze each adjective separately (honest, moral, nice, and likable and talented, resourceful, respectable, and admired).

We recruited the participants through open Polish internet forums and e-mailing lists. We joined numerous publicly accessible internet groups (for example, linked to major Polish cities) where we posted information about a study on perceptions of SH; we asked their users to participate and to snowball this request to their acquaintances. With this information, we provided the participants with a website link to the online study. All of the participants were presented with the materials and filled out the measures in the following order: questions on sex, age and education, one randomly assigned version of the vignette, one randomly assigned measure of victim’s well-being (BDI, HSCL, WHO, or PTSD), and the scale to evaluate the perpetrator. The four questionnaire groups did not significantly differ in terms of age *F* (3, 149) = 0.26, *ns* (*M*_BDI_ = 35.27, *M*_HSCL_ = 37.36, *M*_WHO_ = 36.90, and *M*_PTSD_ = 38.07); male-to-female ratio *χ* ^2^ (3) = 6.38, *ns* (percentage of women for BDI = 85.4, HSCL = 72.5, WHO = 78.6, and PTSD = 72.5); or education *χ* ^2^ (15) = 10.30, *ns*.

## Results

### Perception of the Victim’s Suffering

Due to violation of the parametric assumptions for some of the dependent variables and because of small sample sizes, we decided to conduct a series of Mann–Whitney tests, separately for male and female participants. Similar analysis carried out separately for different age and education groups showed that those two variables did not differentiate significantly between the participants.

We tested the first hypothesis that female victims of SH are perceived to suffer to a greater extent than male victims using a series of Mann–Whitney tests, with the sex of the victim as the independent variable and in subsequent analysis, depression, anxiety, somatic symptoms, and well-being as dependent variables. As the sample sizes for each of the outcome variables were extremely small, we report the exact significance values, rather than the asymptotic ones. As predicted, sex of the victim influenced the perception of their suffering. We observed that all of the scales measuring perceptions of the victim’s state overall showed results consistent with the hypothesis; i.e., a female victim was perceived to suffer more than a male victim. However, men and women differed in the kinds of distress they attributed more to women. Thus, female participants evaluated the female victim as suffering significantly more from depressive symptoms than the male victim on the BDI measure (*U* = 95.50, *z* = −2.16, *p* = 0.03, *r* = −0.35), whereas the male participants evaluated the depressive symptoms to be similar for both male and female victims. However, for the HSCL and WHO-5 questions, the female participants did not distinguish between male and female victims, whereas the male participants did. They perceived the female victim as tending to have more symptoms of anxiety (*U* = 2.00, *z* = −1.96, *p* = 0.06, *r* = −0.65) and more somatic symptoms (*U* = 0.00, *z* = −2.47, *p* = 0.008, *r* = −0.82) as well as worse general well-being (*U* = 0.00, *z* = −2.00, *p* = 0.07, *r* = −0.70) than a male victim. The exact statistics for all effects, together with effect sizes, median, and mean range values are presented in Tables 1 (female participants) and 2 (male participants).

**Table 1:** Perceived suffering of the victim depending on the sex of the victim (study 1)—female participants

	Female victim/male perpetrator *Mdn* (mean range)	Male victim/female perpetrator *Mdn* (mean range)	*U*	*Z*	*r*
Beck Depression Inventory	12.00 (23.63)	6.50 (15.84)	95.50*	−2.16	−0.35
HSCL–anxiety	3.30 (15.39)	2.60 (12.29)	69.50	−1.00	−0.19
HSCL–somatic symptoms	3.50 (14.83)	3.00 (12.96)	77.50	−0.62	−0.11
HSCL–depressive symptoms	3.16 (14.77)	2.58 (13.04)	78.50	−0.56	−0.10
WHO Well-Being Scale	6.00 (17.25)	1.50 (15.25)	105.00	−0.59	−0.10

*p < 0.05 (exact significance)

**Table 2:** Perceived suffering of the victim depending on the sex of the victim (study 1)—male participants

	Female victim/male perpetrator *Mdn* (mean range)	Male victim/female perpetrator *Mdn* (mean range)	*U*	*Z*	*r*
Beck Depression Inventory	13.00 (3.75)	12.50 (3.00)	3.00	−0.49	−0.20
HSCL–anxiety	3.15 (7.00)	1.10 (3.40)	2.00**	−1.96	−0.65
HSCL–somatic symptoms	3.75 (7.50)	2.00 (3.00)	0.00*	−2.47	−0.82
HSCL–depressive symptoms	3.08 (6.75)	1.25 (3.60)	3.00	−1.72	−0.57
WHO Well-Being Scale	8.00 (3.50)	21.50 (7.50)	0.00**	−2.00	−0.70

*p < 0.01 (exact significance)

**p < 0.07 (exact significance)

### Perception of the Perpetrator’s Characteristics

As the distribution of the perception variables was not normal, we conducted a set of Mann–Whitney *U* tests to test the hypothesis that perpetrators of SH on men are evaluated less negatively than perpetrators of SH on women separately for male and female participants. In both analyses, the victim’s (perpetrator’s) sex was introduced as an independent variable, and the following variables were used as dependent measures: honest, moral, nice, likable, talented, resourceful, respectable, and admired. We also checked that the scale filled out as the first measure (BDI, HSCL, WHO, and PTSD) as well as participant’s age or education did not influence further responses.

We found support for our hypothesis in female but not male participants. For the female participants, as expected, the male perpetrator who sexually harassed a female was perceived as significantly less honest, moral, nice, and likable, as well as less respected and admired, than a female perpetrator who sexually harassed a male. The exact statistics for all effects, together with effect sizes, median, and mean range values, are presented in Table 3. However, for the male participants, we found no significant differences in their perception of the perpetrator as a function of his/her sex.

**Table 3:** Perception of the perpetrator, depending on the sex of the victim (study 1)—female participants

	Female victim/male perpetrator *Mdn* (mean range)	Male victim/female perpetrator *Mdn* (mean range)	*U*	*Z*	*r*
Honest	1.00 (47.04)	1.00 (56.52)	1055.00	−2.43*	−0.25
Moral	1.00 (47.01)	1.00 (55.59)	1053.50	−2.83*	−0.28
Nice	1.00 (42.72)	2.00 (56.26)	843.50	−2.49*	−0.25
Likable	1.00 (45.98)	1.00 (56.77)	998.00	−2.29**	−0.22
Talented	4.00 (44.80)	4.00 (51.56)	965.00	−1.22	−0.12
Resourceful	4.00 (45.12)	5.50 (53.30)	975.00	−1.46	−0.14
Respectable	1.00 (43.99)	1.00 (55.99)	900.50	−2.85*	−0.28
Admired	1.00 (46.12)	1.00 (54.47)	1013.50	−1.98**	−0.19

*p < 0.01 (exact significance)

**p < 0.05 (exact significance)

## Discussion

The first study gives overall support to the hypothesis that the suffering of male victims of SH is minimized. Firstly, a male victim is perceived to suffer less than a female victim, although we observe differences between male and female participants in what kind of suffering they attribute to a female victim. According to female participants, a female victim is expected to experience more depression, while the male participants see a female victim as suffering more from anxiety, somatic symptoms, and as having a generally worse well-being following the assault. Secondly, the hypothesis that the female perpetrator of SH on a man is evaluated less negatively than a male perpetrator of SH on a woman is supported in female but not in male participants. A male perpetrator who sexually harasses a woman is seen as less honest, moral, nice, and likable as well as less respected and admired than a male perpetrator who harasses a woman.

While the above results give general support to our hypotheses that male suffering is minimized by both men and women, and that male perpetrators are more negatively evaluated (at least by women), several questions remain. For example, there was a relatively low number of men in our sample, which may explain the failure to obtain some of the predicted results in men. Because of these concerns, in the following study, we sampled a larger number of men. In addition, in study 2, we focused on selected measures of perceived suffering, using five items based on the Beck Depression Inventory to measure perceived depression symptoms and four items from the HCSL to measure perceived somatic symptoms of the victim. We chose the items that distinguished well between male and female victims and had higher response rates, indicating that they were easier for participants to answer (i.e., where large numbers of responses were not omitted by a large number of participants who raised concern that they were unable to imagine an answer to the items).

One interpretation of the above results is that they illustrate a general tendency to perceive male and female actors differently, namely, to see female victims as more fragile than male victims and to see male perpetrators on females in a more negative light than female perpetrators on males. This raises the question are female victims generally perceived as more fragile than male victims and are male perpetrators of assault generally perceived in a more negative light than female perpetrators? In addition, is the minimization of male suffering specific to SH or does it occur in other types of assaults? In the next study, we wanted to verify if this trend is specific to SH or if it is observable in other, non-sexual, types of assault such as financial extortion, thus suggesting that male suffering is generally minimized whatever the kind of assault.

In the second study, we therefore decided to compare perceptions of sexual and financial coercion (extortion) in a similar work setting. Given that extortion is an act of acquiring goods or services through a threat, intimidation, or a different form of pressure (Urdang and Flexner 1969), we can say that SH of the sexual coercion type can be perceived to be a specific form of extortion, as it is an act of acquiring sexual favors by threatening a person’s position in a workplace, and that financial extortion is a form of coercion where the same (dis)incentives are used to acquire money. With that in mind, for the second study, we decided to compare perceptions of victims and perpetrators in comparable cases of sexual coercion (SC) and financial coercion (FC). In both cases, the consequences for the victim were the same, failure to comply with the perpetrator’s demand meant that the victim will not get employed at the company after a period of internship.

**Study 2. The effects of type of coercion and victim’s sex on the perception of victim’s suffering and the perception of the perpetrator**

### Materials and Procedure

The participants in the second study (*n* = 201) were Polish psychology (*n* = 120) and civil engineering (*n* = 81) students with a mean age of 20.26 (SD = 1.32). The sample consisted of 134 women and 57 men, and 10 participants did not state their sex. We collected the data in lecture halls during class; after obtaining the permission from the lecturer, we asked students to fill out the questionnaires at the beginning of their class. As in the first study, the participants were asked to state their sex and age. Next, they were randomly assigned to read one of four vignettes which described an intern. The story used for SH conditions was exactly the same as in study 1; however, as mentioned above, we added two FC conditions. Assuming that SH might be considered to be a type of extortion (“you will get this job if you have sex with me”), the control conditions described the same situation with one sentence changed. Namely, at the end of the internship, the young person was informed by his/her supervisor that s/he can get the job if he pays him/her (“you will get this job if you pay me”). This resulted in the following four conditions: male on female SC, female on male SC, male on female financial coercion (FC), and female on male FC.

After reading the vignette, the participants filled out a number of measures. We used the data from the first study to prepare the tools to measure the perceived depression and somatic symptoms of the victims.

To measure perceived depression, we used five modified items inspired by the Beck Depression Inventory; namely (end of scale items), *S/he is so sad and unhappy that s/he cannot stand it*, *S/he feels irritated all the time*, *S/he has lost all interest in other people*, *S/he believes that s/he looks ugly*, and *S/he has lost interest in sex completely*. Each item was scored on a scale from 0 (*does not have this symptom*) to 3; to obtain the overall depression score, the values were summed. Cronbach’s alpha for the scale was 0.70.

For the perceived somatic symptoms, we used the following four items from the HCSL: [s/he has] *headaches*, *difficulty falling asleep or staying asleep*, *poor appetite*, and [s/he] *feels tense or keyed up*. Each item was scored on a scale from 1 (*not at all*) to 5 (*extremely*), and the average overall score was computed from all the items. Cronbach’s alpha for somatic symptoms was 0.83.

As we wanted to see if the perception of the event by the victim can differ depending on the sex of the victim/perpetrator, we prepared a list of adjectives and their oppositions (*not scary*–*scary*, *not irritating*–*irritating*, and *not flattering*–*flattering*) that were evaluated on a seven-point scale. We asked the participants to rate how they think the event can be perceived by the victim. Exploratory factor analysis showed a three-factor solution. The first factor consists of two items, frightening and threatening (Cronbach’s alpha = 0.57), and it is hereinafter referred to as “scary”; the other two factors are “painful” (five items, e.g., painful, harsh, and unpleasant; Cronbach’s alpha = 0.75) and “offensive” (five items, e.g., offensive and irritating; Cronbach’s alpha = 0.70).

Additionally, as in the previous study, we used a scale to measure perceived communion, agency, liking, and respect of the victim and the perpetrator (Kosakowska 2006). As in the previous study, the Cronbach’s alphas were not satisfactory for the majority of subscales (most of them in the range of 0.65), except for the perceived respect (items, respectable and admired) toward victim (0.78) and perpetrator (0.79). As such, we analyzed separately each of the following items: honest, moral, nice, and likable; talented and resourceful; and a general respectability (mean of two items) score for both the perpetrator and the victim. Moreover, in an open-ended question, we asked the participants to suggest a prison sentence in years for the perpetrator as an indicator of the perceived seriousness of the offense.

Participants were presented with the materials and filled out the measures in the following order: questions on sex and age, one randomly assigned version of the vignette, evaluation of the victim, depression, somatic symptoms, event perception, evaluation of the perpetrator, and prison sentence.

## Results

### Perception of the Victim’s Suffering and Evaluation of the Offense

In order to test whether the perception of the victims’ suffering depended on their sex (hypothesis 1), we conducted two 2 × 2 × 2 (victim’s sex × type of coercion × participant’s sex) ANCOVAs with perceived depression and somatic symptoms as dependent variables and participants’ age and major (civil engineering/psychology) as covariates. We found as predicted that the victim’s sex affected perceptions of the victim’s suffering. Thus, a female victim was evaluated as suffering more both from depression and from somatic symptoms than a male victim. This provides a replication of the major results obtained in study 1. The exact *F* statistics as well as means and standard deviations are presented in Table 4. In addition, unlike study 1, the participants’ sex did not influence the perceived depression and somatic symptoms of the victim; both male and female participants thought a female victim suffered to a larger extent than a male victim.

**Table 4:** Perceived suffering of the victim and perception of the perpetrator of SC or FC depending on the sex of the victim (study 2)

	Female victim/male perpetrator (SD)	Male victim/female perpetrator (SD)	*F*	*d*
Depression	9.25 (2.57)	8.12 (3.01)	*F* (1, 175) = 5.55**	0.40
Somatic symptoms	3.31 (0.90)	3.05 (0.89)	*F* (1, 175) = 4.33***	0.29
Scary	5.58 (1.18)	5.17 (1.22)	*F* (1, 175) = 6.23**	0.34
Painful	5.89 (0.87)	5.17 (1.15)	*F* (1, 175) = 27.03*	0.70
Offensive	6.32 (0.79)	5.81 (1.10)	*F* (1, 175) = 14.83*	0.53
Talented (perpetrator)	3.71 (1.36)	4.05 (1.32)	*F* (1, 172) = 3.43****	0.25
Resourceful (perpetrator)	4.76 (1.83)	5.27 (1.48)	*F* (1, 172) = 3.77***	0.30

*p < 0.001

**p = 0.01

***p < 0.05

****p = 0.06

Further, we wanted to find out if the victim’s sex influences how s/he is perceived to see the offense (hypothesis 3) and the evaluation of the victim. We therefore conducted a series of 2 × 2 × 2 MANCOVAs with sex of the participants, the type of coercion, and the sex of the victim as the independent variables; participants’ age and their major as covariates; and the evaluation of the victim and the perception of the event by the victim as dependent variables. We found no main effects of the victim’s sex on evaluation of the victim with respect to their perceived honesty, morality, niceness, likability, resourcefulness, talent, and general respectability, showing that both the male and the female victims were perceived in the same way on these dimensions of social perception. However, we found a significant effect of the victim’s sex on the perception of the event using Pillai’s trace (*V* = 0.14, *F* (3, 173) = 9.74, *p* < 0.001), and separate ANCOVAs showed that the female victim was perceived as seeing both SC and FC as more scary, as more painful, and as more offensive than the male victim. 1 The exact *F* statistics as well as means and standard deviations are presented in Table 4.

**Fig. 1:**
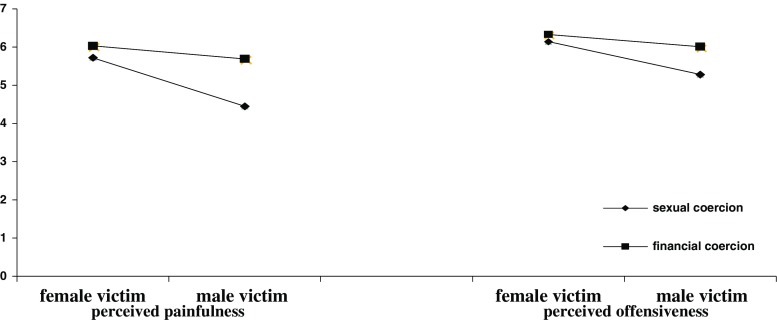
Perceived painfulness (*left side* of the figure) and offensiveness (*right side* of the figure) of the event, interaction effects of the type of coercion and victim’s sex (study 2). Note that the differences are significant for SC and non-significant for FC

There was also a significant interaction effect of victim’s sex and the type of coercion (*V* = 0.5, *F* (3, 173) = 3.08, *p* = 0.02) on the perception of the event as painful. Separate ANCOVAs showed the interaction to be significant for the perception of the event as painful (*F* (1, 175) = 9.01, *p* = 0.003), and there was an interaction effect approaching significance for the perception of the event as offensive (*F* (1, 175) = 3.17, *p* = 0.07). Interestingly, the simple effect analysis showed SC to be more painful and offensive to a female than to a male victim, respectively (*F* (1, 175) = 31.07, *p* < 0.001, *d* = 1.14 and *F* (1, 175) = 14.66, *p* < 0.001, *d* = 0.67), while FC was equally painful and offensive to both a male and a female victim. The interactions are presented in Fig. 1.

### Perception of the Perpetrator’s Characteristics

For the perception of the perpetrator, the only dependent variables that met the requirements to run parametric tests were the evaluation of the perpetrator as resourceful and talented. In order to test whether female perpetrators who assault men are evaluated less negatively than male perpetrators who assault women, we conducted a 2 × 2 × 2 MANCOVA with the type of coercion, victim’s and participant’s sex as independent variables, and perceived talent and resourcefulness as dependent variables, controlling for participants’ age and major. We found a marginally significant effect on the perceived talent and resourcefulness *V* = 0.32, *F* (2, 171) = 2.79, *p* = 0.06. With the use of separate ANCOVAs, we found that the female perpetrator of coercion on a man was seen as more resourceful and marginally more talented. The exact *F* statistics, effect sizes, means, and standard deviations are presented in Table 4. For the evaluation of the perpetrator, we did not find differences between male and female participants.

As the data did not meet the requirements to carry out parametric tests, for the remaining items evaluating the perpetrator as well as the proposed punishment, we used the Kruskal-Wallis test, followed up with Mann–Whitney tests to which we applied a Bonferroni correction; i.e., effects are considered significant only at a 0.025 level of significance. For the perception of the perpetrator as honest, moral, nice and likable, and respectable, the results of the Kruskal-Wallis test showed that the sex of the victim and the type of coercion influence the perception of the perpetrator’s niceness and respectability, respectively, *H* (3) = 10.08, *p* = 0.01 and *H* (3) = 13.34, *p* = 0.004. The follow-up Mann–Whitney test, carried out separately for SC and FC, showed that a man who harasses a woman is less respected (*Mdn* = 1.00, mean range = 41.68) than a woman who harasses a man (*Mdn* = 2.00, mean range = 60.51) *U* = 799.50, *p* < 0.001, *r* = −0.34; no such effect was found in case of FC, and no further significant effects were found for the perceived niceness.

Finally, the suggested prison sentence was influenced by experimental condition *H* (3) = 12.67, *p* = 0.005. The Mann–Whitney test showed that the female perpetrator of SC on a male was given less years of prison (*Mdn* = 1.00, mean range = 39.35) than the male perpetrator of SC on a female (*Mdn* = 3.00, mean range = 59.65) *U* = 703.00, *p* < 0.001, *r* = −0.36, while the sentence given to the perpetrator of FC on a male (*Mdn* = 2.00, mean range = 45.98) and on a female (*Mdn* = 2.00, mean range = 48.96) did not differ significantly, *U* = 1034.00, *ns*, *r* = −0.05.

## Discussion

In the second study, we replicated the major results of the first study, by showing that the female victim of SC by a male is perceived to have more depressive and somatic symptoms than the male victim of a female. Importantly, using a larger sample of men in study 2, we showed that this effect can be detected in male as well as in female participants. We also showed that a female perpetrator of SC on a male is less disrespected than a male perpetrator on a female, as well as perceived as more talented and resourceful. In addition, study 2 showed that SC committed on a woman is perceived to be a more serious offense than SC committed on a man, and is perceived as more scary, painful, and offensive by the victim, and warranting greater punishment for the perpetrator. As such, the second study replicates the major findings of study 1 by showing that women are perceived to suffer more from SC than men. However, it extends the findings of study 1 by showing that women are also perceived to suffer more than men from FC and that people recommend stronger punishments for male perpetrators of opposite-sex SC but not of opposite-sex FC.

Our results therefore answer the three questions raised as a result of the first study. Firstly, are female victims generally perceived as more fragile than male victims? As shown by the main effects of victim’s sex regardless of the type of offense, female victims are perceived to suffer more after both SC and FC than male victims, although they are seen to suffer more and to be more offended than men only in the case of SC. Secondly, is the minimization of male suffering specific to SH or does it occur in other types of assaults? As stated before, the MMS occurs when the male victim is seen as suffering less than the female victim and when the perpetrator of an act on a man is evaluated better that the perpetrator on a woman. This conjunction occurs in our study for both SC and FC. The female victim is seen as suffering more depression and somatic symptoms in both cases and as perceiving the situation as more scary, painful, and offensive. A woman who attacks a man is less disrespected and perceived as more talented, resourceful, and respectable than a man who attacks a woman. The conjunction is visible for SC and the majority of FC variables that were used. As such, the current state of this research leads us to claim that the MMS effect is not specific to assaults that involve sexuality but rather that it might occur for other types of opposite-sex coercion.

Thirdly, are men as perpetrators generally perceived in a more negative light than female perpetrators? Female perpetrators who assaulted a male are seen as more talented, resourceful, and respectable than male perpetrators who assaulted a female. This suggests that as far as perception goes, male perpetrators of opposite-sex coercion are in fact evaluated more negatively than female perpetrators of opposite-sex coercion. There are also differences in the punishment suggested for male and female perpetrators depending on the act that they committed. A woman who sexually harassed a man is judged to deserve a lower prison sentence than a man who sexually harassed a woman, while the punishment is the same for male and female perpetrators of FC. Nevertheless, for the financial and sexual coercion, in terms of social perception, men as perpetrators are perceived more negatively than women as perpetrators.

## General Discussion

We put forward the hypothesis that male suffering is minimized in two ways: (1) through perception of the male victims as less affected by the act of SH than female victims and (2) through perceiving of perpetrators of SC on men in a better light than perpetrators of SC on women. These main hypotheses were based on previous research regarding both actual victims of SH and the social perception of victims of SH. Research on victims of SH shows that men suffer to the same extent as women as a result of this offense (Settles et al. 2011; Birkeland et al. 2010; Street et al. 2007a, b; Vogt et al. 2005; Magley et al. 1999), while the studies on the perception of the victims show the men to be seen as less influenced by SH (LaRocca and Kromrey 1999; Berdahl et al. 1996; Struckman-Johnson and Struckman-Johnson 1993; McKinney 1992; Konrad and Gutek 1986).

Our research extends the earlier studies by looking in more detail at the perceived suffering of the victims, as we used variables usually used in research on actual victims of SH, such as depression and somatic symptoms, and by considering the effect of sexual harassment on the characteristics attributed to the victim and perpetrator, on how the victim evaluated the event, and on what punishment was recommended for the offense. What is also unique about the presented studies is that we compared the perception of SH victims to victims of a different type of coercion (i.e., financial extortion). The results of the two studies are very straightforward and support the hypothesis that male suffering is minimized in two ways (perception of victim’s distress and perception of the perpetrator’s characteristics), not only when it is caused by SC but also when it entails financial extortion. Those studies show that people perceive a man harassed by a woman to suffer less than a woman harassed by a man and that a woman who harasses a man is evaluated less negatively than a man who harasses a woman. Further work needs to be done to clarify whether male victims are seen as suffering less due to a general stereotype that a woman is unable to hurt a man or because the situation of a female on male SH is perceived to be more of a joke than an actual threat. Finally, another limitation of our studies is that they only investigated opposite-sex coercion. Studies investigating both same- and opposite-sex SH should bring further clarifications of the role of gender stereotypes in producing the MMS phenomenon.

While the results we have obtained build a wider picture of the perception of the attributes of SH victims and their perpetrators, they are not altogether surprising. Through gender stereotypes, men learn to be tough and not to show their weakness. Unlike countries like the USA or the UK where the social revolution started after World War II, the stereotype of a “tough guy” is probably even stronger in a society like Poland. As shown by the results of cross-cultural studies (Koopman et al. 1999), Poland is much lower in a gender egalitarianism raking (mean score = 4) than England (mean score = 14). Moreover, according to World Economic Forum’s report on gender gaps (Hausmann et al. 2012), Poland places 53 among 135 countries, while the UK ranks 18 and the USA 22. This is probably because in Poland, gender-stereotype changes did not start happening until after the fall of the Berlin wall and the image of a strong, macho-like man persists. As the men do not explicitly show their feelings in everyday life, the society expects them not to experience sadness, somatizations, or depression and judges them accordingly. Quite possibly, if we asked the participants to evaluate the perceived anger, the male victim might be seen to be more angry, as expression of this emotion is consistent with the male gender stereotype.

On the other hand, one might wonder why the perception of the female perpetrator is less negative than the perpetrator of the male perpetrator. Previous research showed (e.g., Eagly and Karau 2002) that women acting in a gender-inconsistent fashion in the workplace, for example, by being assertive, are evaluated in worse light than men who are being assertive. As such, one might expect that a woman behaving in a way more fitting for a man, i.e., a woman who sexually harasses an intern, would be evaluated *more* negatively than a man who does the same, as the man’s behavior may seem less gender-inconsistent.

More studies need to be carried out in order to better establish whose sex more strongly influences the perception of victims and perpetrators of SH—the victim’s or the perpetrator’s. We believe that further studies should concentrate on same-sex SH as well as more complex descriptions of SH cases. While we acknowledge that SH is a problem that affects more women than men, we believe that the men who become victims of SH deserve the same kind of compassion, understanding, and justice. This is why we think it is important to find out what influences how they are perceived and how the society judges their oppressors.

## Footnotes

1 We also found a significant main effect of the type of offense, which showed SC to be seen as less painful and less offensive than FC. This result is consistent with another study (unpublished) carried out comparing SH to FC, where FC is seen as more abusive offense than SH across different dependent variables. Both SH and FC were perceived by the participants to be equally likely to occur; as such, we do not fully understand the source of this result. Moreover, we found the sex of the participants to influence the perception of respect, talent, and resourcefulness of the victim, such that women evaluated the victim higher than men, and for the evaluation of the event as scary and offensive, such that women though the event to be more offensive and scary. However, this results are not our main point of interest; thus, we do not discuss them further.
